# Association Between Sleep Quality and Temporomandibular Disorders (TMD) Symptoms Among Adults: A Cross-Sectional Study

**DOI:** 10.7759/cureus.87818

**Published:** 2025-07-13

**Authors:** Abali Wandala, Naila Ikram, Maryam Binte Safiullah, Azeem Hussain Soomro, Yashar Mashayekhi, Amal M Nawasrah, Aisha Ali, Nayyab Tariq, Rida Malik, Shehrezad Jamshed, Mishal Fatima

**Affiliations:** 1 Interventional Cardiology, Mission Vascular and Vein Institute, Mission, USA; 2 Medicine, Universidad Adventista del Plata, Libertador San Martín, ARG; 3 Dentistry, Shifa College of Dentistry, Rawalpindi, PAK; 4 Oral Pathology, Dow University of Health Sciences, Karachi, PAK; 5 Medicine, Leicester University Hospital, Leicester, GBR; 6 Prosthodontics, College of Dentistry, Imam Abdulrahman Bin Faisal University, Al Khobar, SAU; 7 General Dentistry, Ziauddin University, Karachi, PAK; 8 Dentistry, Foundation University College of Dentistry and Hospital, Islamabad, PAK; 9 General Dentistry, Shifa College of Dentistry, Rawalpindi, PAK; 10 Dentistry, MindWave Research Center, Islamabad, PAK

**Keywords:** adult population, fonseca anamnestic index, pittsburgh sleep quality index, psychosocial factors, sleep quality, temporomandibular disorders

## Abstract

Background: Temporomandibular disorders (TMDs) are a cluster of musculoskeletal and functional disorders of the jaw joint and associated muscles, frequently associated with pain, stress, and decreased quality of life. More recent literature emphasizes a strong correlation between symptoms of TMD and poor sleep quality, but the literature is limited in diverse adult populations. This study explores the interaction between sleep quality and TMD symptoms, as well as the demographic variables of age, gender, and marital status.

Methods: We enrolled 385 adults in this cross-sectional study from both general and dental outpatient departments in Islamabad, Pakistan. To assess TMD symptoms, we used the Fonseca Anamnestic Index (FAI), and we measured sleep quality using the Pittsburgh Sleep Quality Index (PSQI). Data collection was conducted between February 2025 and April 2025. Data were analyzed using IBM Corp. Released 2017. IBM SPSS Statistics for Windows, Version 26.0. Armonk, NY: IBM Corp., including descriptive statistics, Spearman correlation, Mann-Whitney U tests, Kruskal-Wallis H tests, and ordinal logistic regression.

Results: The total sample (N = 385) consisted of 182 men (47%), 150 women (39%), and 53 participants (14%) who did not wish to have their gender revealed. The largest age group was 18-25 years (N = 275; 71%). There was a strong positive association between PSQI and FAI scores (r = 0.223, p-value < 0.001), indicating that lower sleep quality was associated with more severe TMD symptoms. Participants who were not taking medication (N = 106, 28%) reported worse scores than those taking medication (N = 279, 72%). The regression analysis indicated that TMD severity was substantially predicted by PSQI scores (B = 0.119, p-value < 0.001).

Conclusion: This research highlights a significant association between poor sleep quality and heightened TMD symptom severity in adults. Demographic variables, such as age, gender, and marital status, also influenced these results. These results suggest the utility of sleep evaluation in diagnosing TMD and the development of multidisciplinary treatment protocols. The use of a longitudinal research design that incorporates objective sleep assessment and psychological measures is recommended for future studies to examine causal pathways.

## Introduction

Temporomandibular disorders (TMD) refer to a group of conditions where pain or problems with movement are found in the temporomandibular joint as well as the muscles and tissues around it. Very often, these health problems are caused by many factors and can seriously affect both daily life and quality of life [1]. Temporomandibular disorders (TMD) are difficult to diagnose and manage due to their multifactorial causes, including bruxism, hormonal factors, and psychological stress, and their symptoms overlap with other orofacial pain conditions [2,3]. Diagnosis is further complicated by similarities between TMD, trigeminal neuralgia, and other neuropathic facial pain syndromes, often leading to misdiagnosis [4]. Therefore, conservative, non-surgical treatments such as occlusal splints, physiotherapy, stress management, behavioral therapy, and pharmacological interventions (e.g., nonsteroidal anti-inflammatory drugs [NSAIDs], muscle relaxants) are generally preferred and have been shown to be effective in symptom relief [5]. Temporomandibular disorders impact as many as 15% of adults and can result from a mix of medical and lifestyle causes. Reliable diagnosis depends on the diagnostic criteria (DC)/TMD criteria, and noninvasive treatment by a multidisciplinary team is the best way to help most of these patients [6].

New findings now classify temporomandibular disorders (TMD) as biopsychosocial issues, which means doctors tend to focus on diagnosing and using gentle therapies. Even with increased knowledge, lots of treatments lack proper research support, which points to the need for care based on evidence [7]. TMD belongs to a group of complex conditions that often show a large placebo effect, which affects how we interpret therapy results. For now, reversible therapies are recommended before irreversible treatments, because the latter require new research and the use of standard tools to measure results [8]. Sometimes joint or muscle pain caused by TMD can mimic chronic rhinosinusitis (CRS), leading doctors to confuse the diagnosis. Since many people with TMD have pain in the face and sinuses, it is significant to distinguish TMD from CRS to give proper treatment [9].

Although it is extensively utilized in the study of the topic of sleep, the term sleep quality is an ill-defined concept. One study highlighted the challenge when objectively correlating this subjective experience and surmised constraints in the widely employed methodologies in assessing this aspect, including polysomnography, actigraphy, and cyclic alternating patterns, and theorized on multidimensional and personalized assessment measures [10].

Studies demonstrate that people with TMD and pain experience much worse sleep quality than those without any diagnosis. Having chronic TMD is closely connected to sleep problems, so doctors should assess a patient’s sleep when managing TMD [11]. A detailed analysis confirmed that those with TMD report poor subjective sleep quality, but the relationship is most significant for muscle and joint pain in TMD cases. A poor-quality sleep pattern in patients was linked to a 4.45 times higher risk of TMD, showing it is important to assess sleep for people with the disorder [12].

TMD is reported in about 41.8% of patients, and its severity can strongly affect how patients feel, think, and sleep. Of note, 69.6% of people with TMD experienced trouble sleeping, which points to its close link with mental and physical health [13]. As symptoms of TMD get worse, it becomes harder to sleep well, mainly in individuals who have pain-related and intra-articular forms. Patients who experienced combined pain reported worse sleep, and pain-related TMDs were linked to threefold greater odds of poor sleep [14].

Understanding how TMD symptoms are linked to sleep quality allows us to offer better treatment and guide patients more effectively. Yet, despite more interest in this field, we still need additional studies, especially among various adult groups. This investigation aims to identify whether sleep quality is related to TMD symptoms in adults. The main aim is to show how sleep disruptions may influence TMD and to promote the idea that managed sleep disturbances should play a role in TMD management.

Rationale

Temporomandibular disorders (TMD) involve several conditions that bring pain and problems to the jaw joint and the muscles used for jaw movements. They can get in the way of normal daily living and lower a person’s quality of life. In general, getting good sleep is connected to overall health and can be influenced by physical pain, stress, and other illnesses. Some existing research has looked at whether there is a connection between TMD symptoms and sleep problems, and it seems there could be. Still, research on this subject has been small-scale, targeted at populations or differing in their culture and healthcare context. Because of this, the findings could only be applied to adults with these experiences. The research is designed to uncover the relationship between sleep and TMD symptoms in adults. This research considers a broad group of adults and analyzes them using regular assessment tools, aiming to provide a better view of the link between these two situations. Results may help improve TMD management methods for patients.

Primary objective

The primary objective of this study is to examine the relationship between sleep quality and symptoms of temporomandibular disorders (TMD) in adults.

Secondary objectives

The secondary objectives of this study include assessing the prevalence of poor sleep quality among individuals with specific TMD symptoms, investigating the type and severity of TMD symptoms experienced by the participants, and evaluating the influence of socio-demographic factors, such as age, gender, and marital status, on the relationship between sleep quality and TMD symptoms.

## Materials and methods

Study design and methods

A cross-sectional study design was used to explore the relationship between sleep quality and TMD symptoms in adults. To create differences in how participants slept and expressed symptoms, the researchers included subjects from various backgrounds and ages. To carry out the study in Islamabad, Pakistan, dental outpatient departments and general health clinics were brought in. Despite the presence or absence of recognized sleep problems, these venues included adults who had reported TMD complaints. Finding results among candidates from several clinical areas also extended their reliability to the adult community. 

Using the chosen research design, researchers were able to obtain details on age, gender, and past medical history. Standardized instruments were used to evaluate TMD symptoms and sleep quality. 

Sample size and technique

The infinite population formula was employed to calculate the sample size for this research since the actual population of people was not known. The formula employed is:

\[n = \frac{Z^2 \cdot p (1 - p)}{d^2}\]

In this formula, Z is the z-score relating to the required confidence level, p is the projected prevalence or proportion, and d is the acceptable margin of error. For a 95% confidence interval, Z is set at 1.96; the margin of error (d) is constant at 0.05. Expected prevalence (p) was derived from a prior study done in Pakistan with a similar population, which found a frequency of 40.7%. Hence, p was decided to be 0.407. Using these numbers, one could estimate a necessary sample size of 385 participants [15].

Participants were recruited using a convenience sampling method. Eligible patients were approached consecutively as they visited these clinics during the study period. This approach allowed for a diverse sample representing different backgrounds and experiences with TMD symptoms and sleep disturbances.

Inclusion criteria

Participants who were suitable for this research were adults above the age of 18 years. Only those patients presenting with a single or multiple signs of temporomandibular disorders (TMD) were eligible for consideration. Participants also needed to provide informed consent willingly and to be capable of completing the study questionnaire. These conditions guaranteed that the study population had individuals directly applicable to the research target and also capable of fully participating in the data collection.

Exclusion criteria

Participants were excluded from the study if they had a history of previous trauma or surgery to the jaw, as these types of conditions would confound the evaluation of TMD symptoms. Patients with established sleep disorders not related to TMD were also excluded to avoid interference with the individual’s objective of interest in TMD-related symptoms. In addition, individuals who are now diagnosed with psychiatric conditions or are on sedative medication were excluded, considering the possible effect that these elements may exert on symptom reporting and pain sensitivity. Finally, those who have systemic illnesses that are known to compromise pain sensitivity or jaw movement were excluded to reduce variability due to intrinsic medical conditions.

Data collection tools and procedures

A structured questionnaire was prepared with three major parts: demographic details, sleep quality assessment, and evaluation of temporomandibular disorder (TMD) symptoms. Relevant background characteristics were incorporated into the demographic section using questions developed by the authors. In the other two sections, well-established and validated questionnaires were used, including the Pittsburgh Sleep Quality Index (PSQI) to measure sleep quality and the Fonseca Anamnestic Index (FAI) to measure TMD symptoms. It is essential to note that the FAI is used to assess the symptoms of TMD, including jaw pain, reduced jaw movement, and associated headaches; however, it does not serve as a diagnostic tool for TMD [16]. The original English versions of both tools were used in this study. As all participants had a basic understanding of English, no translation or cultural adaptation was required. However, for participants who needed assistance in understanding any item, trained staff provided verbal guidance to ensure clarity without influencing their responses. The researchers integrated original and established questions, thus coming up with a complete and valid sample of data corresponding to the objectives of the study.

Demographic information

In the first part of the questionnaire, demographic information was collected to evaluate possible relationships between the individual features and the symptoms of temporomandibular disorder (TMD), together with the quality of sleep. This section of a survey was specially designed by the authors according to the current study and contained the items about age, gender, marital status, level of formal education, occupation, chronic illnesses, prescriptions, tobacco or smoking, and use of caffeine. Marital status was considered due to its potential impact on psychosocial factors such as stress and social support, which can influence both sleep quality and the severity of TMD symptoms, as indicated by previous research on psychosocial influences on health outcomes. These factors allowed the complete description of the study population and the making of subgroup comparisons to get a better idea of how demographic parameters could affect the extent of TMD symptoms and sleep disturbances (Appendix).

Pittsburgh sleep quality index (PSQI)

To get an idea of sleep quality, the Pittsburgh Sleep Quality Index (PSQI) was used. Developed by Daniel J. Buysse and others in 1989, the PSQI is a survey of 19 questions that anyone can fill out on their own. Seven component scores are created from these items, and all scores are combined to obtain a global score of 0 to 21. Getting a higher score from the questionnaire means that sleep quality is worse. Each item can be identified by a score of 0, 1, 2, or 3. A Cronbach’s alpha of 0.83 demonstrates that the PSQI is internally reliable [17]. The original author granted due permission for the scale to be used in this study.

Fonseca anamnestic index (FAI) 

We used the Fonseca Anamnestic Index (FAI) to assess symptoms of temporomandibular disorders (TMD). In 1992, Dr. Dickson da Fonseca developed the FAI. It consists of 10 items and can be completed alone by patients with TMD to indicate jaw pain, strange sounds in the joints, reduced jaw opening, headaches, and stress. All questions are answered using "Yes" (scored 10 points), "Sometimes" (5 points), or "No" (0 points), and the final score helps medical professionals determine if TMD is mild, moderate, or severe. Good reliability for screening is demonstrated by the FAI, with Cronbach’s alpha coefficients between 0.684 and 0.692 [18]. Due permission was granted by the original author for the scale to be used in this study.

Data were gathered using self-administered questionnaires, which participants completed independently at general health clinics and dental outpatient departments. Trained staff were available to provide instructions or assistance if needed, and in cases where participants faced difficulties due to low literacy, staff provided verbal guidance to help them understand the questions. However, no formal interviews were conducted, and participants responded on their own. All participants provided written informed consent before participation.

Statistical analysis

IBM Corp. Released 2017. IBM SPSS Statistics for Windows, Version 26.0. Armonk, NY: IBM Corp., which was used to input and analyze the data. Both descriptive and inferential statistical processes were used. The demographic and clinical characteristics of the participants were explained with frequencies and percentages. Inferential techniques were applied to determine how the study variables are related and different from each other. 

A Spearman correlation analysis was performed to test the association between the scores of the Pittsburgh Sleep Quality Index (PSQI) and those of the Fonseca Anamnestic Index Questionnaire (FAIQ). To compare scores on the PSQI and FAIQ among those currently taking medication, the Mann-Whitney U-test was used. The Kruskal-Wallis H test was used to analyze the comparison between PSQI and FAIQ scores in distinct groups based on age, gender identity, and marital status. Ordinal logistic regression analysis was performed to investigate whether the PSQI scores significantly predicted the level of TMD symptoms according to FAIQ. Regression model normality was evaluated by running a standard probability plot of regression residuals. Lastly, chi-square tests were conducted to examine relationships between categorical variables, including age, gender, and marital status. A statistical significance level of p < 0.05 was chosen. The use of this method made it possible to explore the data fully and how each study variable relates to the others.

Ethical considerations

This research was carried out according to ethical research standards for the protection, dignity, and rights of all the participants. Ethical approval was granted by the Institutional Review Board of MindWave Research Centre, Islamabad, Pakistan, before the study commenced (IRB-2025-0058). The participants were given adequate information concerning the reason for conducting the study and the processes, as well as their rights, both verbally and through an informed consent form. Voluntary participation in the research was required. Each participant provided written informed consent before data collection. Participant confidentiality was ensured at all costs; all data were anonymized and utilized for research purposes only. Participants were made aware of their right to withdraw from the research process at any point without prejudice. No physical, psychological, or legal harm was inflicted on the participants by taking part in the research. Data collection took place from February 2025 to April 2025.

## Results

Table 1 illustrates the demographic characteristics of 385 study subjects. The majority of participants were young adults between the ages of 18 and 25 years (n = 275, 71%), with a significant proportion in the second age bracket, 26-35 years (n = 95, 25%) and 36-45 years (n = 15, 4%). Concerning gender, 182 respondents (47%) were males, 150 (39%) were females, and 53 (14%) did not want to reveal their gender. There were 162 (42%) married, 107 (28%) single, 90 (23%) divorced, and 26 (7%) widowed participants based on the status among the respondents.

**Table 1:** Demographic characteristics of participants (N=385) f: Frequency, %: Percentage, TMD: Temporomandibular disorders

Variable	f	%
Age	-	-
18-25 years	275	71
26-35 years	95	25
36-45 years	15	4
Gender	-	-
male	182	47
female	150	39
prefer not to say	53	14
Marital status	-	-
single	107	28
married	162	42
divorced	90	23
widowed	26	7
Educational level	-	-
primary	98	26
secondary	145	38
Higher secondary	99	26
Graduate/bachelor’s	33	9
postgraduate	10	3
Occupation	-	-
student	117	30
employed	103	27
unemployed	135	35
retired	30	8
Type of chronic illness	-	-
hypertension	92	24
diabetes	124	32
cardiovascular disease	88	23
sleep disorder	61	16
TMD (temporomandibular disorders)	11	3
other	9	2
Currently taking medication	-	-
Yes	279	72
No	106	28
Tobacco or smoking product usage	-	-
Yes, regularly	137	36
occasionally	157	41
no	83	22
used to, but quit	8	2
Caffeinated drinks consumption	-	-
Yes, regularly	161	42
occasionally	158	41
no	66	17

Regarding the level of education, secondary school graduates comprised the largest proportion of the population (n = 145, 38%), followed by primary school graduates (n = 98, 26%) and higher secondary school graduates (n = 99, 26%). Fewer of them had a bachelor's degree (n = 33, 9%) or a postgraduate degree (n = 10, 3%).

Occupationally, there were 135 (35%) unemployed individuals, 117 (30%) students, 103 (27%) employed individuals, and 30 (8%) retirees. Other chronic medical conditions included diabetes, which had 124 participants (32%); hypertension, which had 92 (24%); cardiovascular disease, which had 88 (23%); and sleep disorders, which had 61 (16%). Eleven participants (3%) reported temporomandibular disorder (TMD), and 9 (2%) reported other conditions. There were 279 (72%) participants on medication during the time of studying, and 106 (28%) others without any medication indicated. The frequency of smoking or being exposed to tobacco or smoking products was normal in 137 (36%), occasional in 157 (41%), absent in 83 (22%), and reported but stopped in 8 (2%). In terms of intake of caffeine, 161 individuals (42%) used caffeinated drinks habitually, 158 (41%) did it intermittently, and 66 (17%) never did.

Table 2 shows the findings of a correlation analysis of the Spearman, which evaluates the correlation between sleep quality, the Pittsburgh Sleep Quality Index (PSQI), and temporomandibular disorder (TMD) symptoms, the Fonseca Anamnestic Index questionnaire. The results showed that the PSQI and Fonseca scores correlated positively (r = 0.223, p-value < 0.001) and were statistically significant, which implies that the worse the individual’s sleep, the more likely they are to experience TMD symptoms. Even though the parametric correlation value is rather low, looking at the significance value, it can be concluded that there is a continuous correlation between the sleep disturbances and the extent of temporomandibular dysfunction symptoms amongst the sample. This observation proves the possible connection between sleep quality and orofacial health and underlines the necessity to pay specific attention to treating sleep problems in patients with TMD-related complaints.

**Table 2:** Spearman’s correlation between Pittsburgh Sleep Quality Index and Fonseca’s Anamnestic Index questionnaire **: p<0.001 considered significant; r: Correlation coefficient

Variable	r	p
Pittsburgh Sleep Quality Index & Fonseca’s Anamnestic Index Questionnaire	0.223	<0.001^**^

Table 3 gives the results of the Mann-Whitney U-test on (a) Pittsburgh Sleep Quality Index (PSQI) scores between the subjects taking medication and those not taking medication, and (b) Fonseca's Anamnestic Index questionnaire scores between the subjects taking medication and those not taking medication. The result shows that both PSQI and Fonseca scores differed significantly in terms of medication status. There was a higher mean rank on the PSQI (mean rank = 213.14, p-value = 0.028) and Fonseca scale (mean rank = 215.00, p-value = 0.024) among the participants not taking medication, indicating that they perceived low sleep quality and high severity of TMD symptoms compared to the participants who were taking medication. These findings suggest that the use of medications may be related to improved sleep and reduced TMD symptoms, indicating a possible moderating role of pharmacological treatment in these two areas.

**Table 3:** Mann–Whitney U-test comparing participants that are currently taking any medication, either yes or no, on the Pittsburgh Sleep Quality Index and Fonseca’s Anamnestic Index questionnaire scores N: Number of participants, U: Mann–Whitney U statistic, Z: Standardized test statistics, **: p<0.01 considered significant

Variable	Currently Taking Any Medication	N	Mean rank	U	Z	p
Pittsburgh Sleep Quality Index	Yes	279	185.35	12652	-2.193	0.028^*^
-	No	106	213.14	-	-	-
Fonseca’s Anamnestic Index Questionnaire	Yes	279	185.00	12587	-2.250	0.024^*^
-	No	106	215.00	-	-	-

Table 4 provides details of the Kruskal-Wallis H test comparing Pittsburgh Sleep Quality Index (PSQI) and Fonseca Anamnestic Index Questionnaire scores across three age groups. The quality of sleep, as measured by the PSQI mean ranks, was also statistically significant among different age groups (H = 18.323, p-value < 0.001). Older respondents (36-45 years) had higher mean ranks of PSQI, and thus lower sleep quality compared to those who were younger. Similarly, Fonseca scores also differed significantly across age groups (H = 7.245, p-value = 0.027), indicating that the severity of TMD symptoms increased with age. These results suggest that sleep disturbance, as well as TMD symptoms, are likely to increase among older age groups within the sampled research participants.

**Table 4:** Kruskal–Wallis H-test comparing Pittsburgh Sleep Quality Index and Fonseca’s Anamnestic Index questionnaire scores by age N: Number of participants, H(x2): Kruskal–Wallis test statistic, df: Degree of freedom; **: p<0.01 considered significant

Variable	Age	N	Mean rank	H(x^2^)	df	p
Pittsburgh Sleep Quality Index	18–25 years	275	180.14	-	^-^	-
-	26–35 years	95	215.34	18.323	2	<0.001^**^
-	36–45 years	15	287.23	-	-	-
Fonseca’s Anamnestic Index Questionnaire	18–25 years	275	185.00	-	^-^	-
-	26–35 years	95	215.00	7.245	2	0.027^*^
-	36–45 years	15	260.00	-	-	-

Table 5 reveals considerable gender disparities in terms of sleep quality and temporomandibular disorder (TMD) symptoms. The Kruskal-Wallis H test showed a significant difference in different gender groups about the Pittsburgh Sleep Quality Index (PSQI) (H=14.227, df=2, p-value=0.001), with females having the highest mean rank (241.39), followed by males (196.03) and those who preferred not to indicate their genders (176.41), meaning a poorer sleep quality in females. Likewise, there was also a significant difference in the Fonseca Anamnestic Index scores (H = 5.844, df = 2, p value = 0.050), where females indicated the highest number of mean ranks (217.29), indicating that females might have higher TMD symptom severity than males and those who did not wish to specify their gender.

**Table 5:** Kruskal–Wallis H-test comparing Pittsburgh Sleep Quality index and Fonseca’s Anamnestic Index questionnaire scores by gender N: Number of participants, H(x2): Kruskal–Wallis test statistic, df: Degree of freedom; **: p<0.01 considered significant

Variable	Gender	N	Mean rank	H(x^2^)	df	p
Pittsburgh Sleep Quality Index	Male	182	196.03	-	^-^	-
-	Female	150	241.39	-	-	-
-	Prefer not to say	53	176.41	14.227	2	0.001^**^
Fonseca’s Anamnestic Index Questionnaire	Male	182	200.52	-	^-^	-
-	Female	150	217.29	-	-	-
-	Prefer not to say	53	179.73	5.844	2	0.05^*^

Table 6 presents the results of the Kruskal-Wallis H test conducted on sleep quality and the intensity of TMD symptoms in different groups based on marital status. The mean ranks of marital status place the widowed at the highest, resulting in the worst PSQI scores, followed by divorced individuals, with a statistically significant difference in PSQI scores across marital status categories (H = 9.224, p = 0.026). Likewise, the Fonseca scores differed dramatically by marital status (H = 8.729, p-value = 0.033), such that divorced and widowed respondents presented an increased score regarding TMD symptoms compared to those who were single or married. These observations indicate that divorce or widowhood could be linked to increased disturbances of sleep and more serious TMD-related symptoms.

**Table 6:** Kruskal–Wallis H-test comparing Pittsburgh Sleep Quality Index and Fonseca’s Anamnestic Index questionnaire scores by marital status N: Number of participants, H(x2): Kruskal–Wallis test statistic, df: Degree of freedom; **: p<0.01 considered significant.

Variable	Marital status	N	Mean rank	H(x^2^)	df	p
Pittsburgh Sleep Quality Index	Single	107	170.88	-	^-^	-
-	Married	162	193.94	9.224	3	0.026^*^
-	Divorced	90	205.21	-	-	-
-	Widowed	26	235.92	-	-	-
Fonseca’s Anamnestic Index Questionnaire	Single	107	175.00	-	^-^	-
-	Married	162	188.00	8.729	3	0.033^*^
-	Divorced	90	225.00	-	-	-
-	Widowed	26	235.00	-	-	-

Table 7 presents the results obtained through ordinal logistic regression, which indicate that Pittsburgh Sleep Quality Index (PSQI) scores were significantly associated with an increased severity of TMD, as determined by the Fonseca Anamnestic Index. In particular, the unit increment of PSQI was associated with a higher likelihood of experiencing more severe TMD symptoms across lines (B = 0.119, p-value of < 0.001). The moderate standardized coefficient (0.207) and the 95% confidence interval (0.063-0.175) indicate a consistent and significant relationship. These findings are statistically conclusive, indicating a positive correlation between poor sleep quality and reports of severe TMJ symptoms, which lends clinical validity to the analysis of sleep disturbances in patients with or without temporomandibular disorders.

**Table 7:** Ordinal logistic regression predicting higher levels of severity based on Fonseca’s Anamnestic Index and the Pittsburgh Sleep Quality Index B: Coefficient, S.E: Standard error, β: Standardized coefficient, LL: Lower limit, UL: Upper limit; CI: Confidence interval, **: p<0.01 considered significant.

Variable	B	95% Cl	-	S.E	β	P
-	-	LL	UL	-	-	-
Constant	9.887	7.142	12.63	1.396	-	<0.001^**^
Pittsburgh Sleep Quality Index	0.119	0.063	0.175	0.029	0.207	<0.001^**^

**Figure 1:**
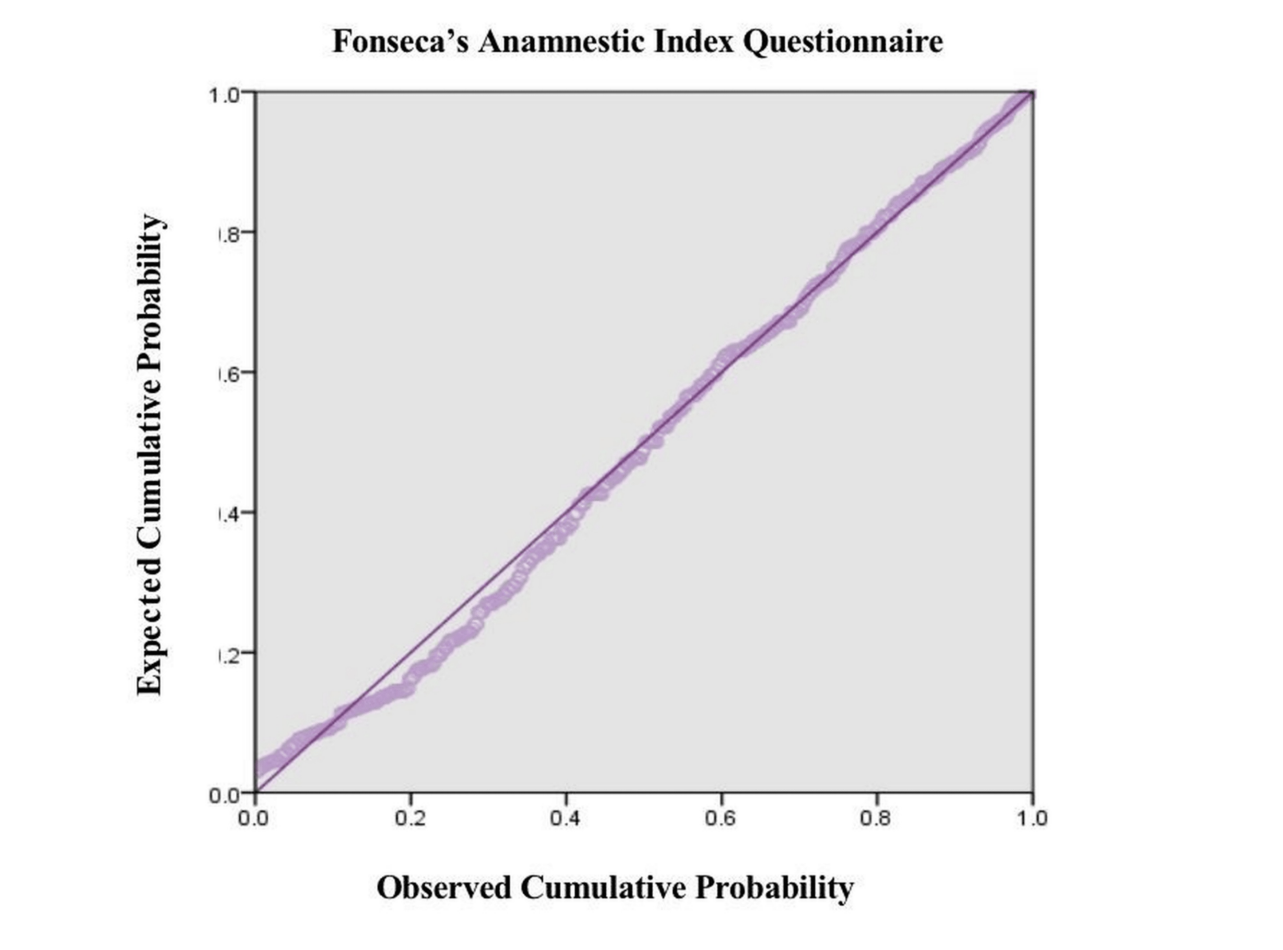
Standard probability plot of regression standardized residuals for Fonseca's Anamnestic Index questionnaire

Figure 1 illustrates a normal probability plot of regression standardized residuals of Fonseca's Anamnestic Index Questionnaire scores. The points trend closely along the diagonal reference line, which means that the residuals are nearly normally distributed. This validates the normality assumption in the regression model, implying that the analysis's findings are statistically sound.

Table 8 shows the results of chi-square tests to evaluate the significance of gender and marital status distribution across various age groups. Relationships with age and gender showed a statistically significant relationship (χ² = 31.6, p-value < 0.001), suggesting that gender distribution differed markedly across age groupings. Particularly among those aged 18 to 25, most of the respondents identified as either male (n = 147) or female (n = 106), with just a small number (n = 22) choosing "prefer not to say." In contrast, those in the 26-35 and 36-45 age groups showed a more balanced distribution among all gender categories, including a rather larger proportion of those who preferred not to disclose their gender.

**Table 8:** Descriptive statistics of demographic variables (age, gender, marital status) f: frequency; %: percentage; p: level of significance; p-values calculated using the chi-square test; **: p<0.01 considered significant.

Variables	f	Male	Gender Female	I prefer not to say	p	x^2^	Single	Married	Marital status Divorced	Widowed	p	x^2^
Age	-	-	-	-	<0.001^**^	31.6	-	-	-	-	0.002^**^	20.6
18-25 years	275	147	106	22	-	-	88	116	58	13	-	-
26-35 years	95	30	39	26	-	-	17	43	24	11	-	-
36-45 years	15	5	5	5	-	-	2	3	8	2	-	-

Age and marital status also showed a meaningful correlation (χ² = 20.6, p value = 0.002). While participants in older age groups, especially the 26-35 and 36-45 age categories, showed growing rates of divorced and widowed status, those in the youngest age group (18-25 years) were more likely to be single (n = 88) or married (n = 116). For younger age groups, there were relatively few divorced (n = 8) or widowed (n = 2) participants among those aged 36 to 45 years. Overall, the data pointed to notable demographic trends; both gender identity and marital status markedly vary across age levels, which could affect or correlate with other research variables.

## Discussion

The purpose of this research was to investigate the association between sleep quality and temporomandibular disorder (TMD) symptoms in a multicultural adult population, employing the Pittsburgh Sleep Quality Index (PSQI) and Fonseca's Anamnestic Index Questionnaire (FAIQ). Our results showed a positive correlation between TMD severity and poor sleep quality, as also indicated by earlier studies with increased PSQI scores in TMD patients. This confirms that worsening TMD symptoms are related to more sleep disturbances and emphasizes the importance of considering sleep quality when assessing TMD [19].

In the present study, participants who were currently taking medication reported better sleep quality than those who were not. This result aligns with evidence suggesting that medication can disrupt the intricate balance of neurotransmitters that regulate sleep and wakefulness. Such neurochemical actions may partly account for why medication use impacts the sleep quality of temporomandibular disorder patients [20]. The present study found that participants who used medication reported fewer intensive TMD symptoms than non-users. This implies that there might be a correlation between the consumption of drugs and less TMD-associated pain. These results align with previous literature demonstrating the widespread application of analgesics and other drugs in the treatment of TMD, although the overall evidence for their efficacy remains limited [21].

According to the present research findings, the quality of sleep significantly declines with age, with the oldest age group experiencing the poorest sleep. This aligns with other studies, which have shown a direct relation between advancing age and poor sleep quality. The replication across studies points to age as a critical factor that affects sleep health among adults [22]. Our findings showed a statistically significant increase in TMD symptom severity with advancing age. This pattern generally aligns with earlier longitudinal research, which indicates a rising incidence of TMD with increasing age. Additionally, our cross-sectional design might restrict the ability to identify genuine age-related risk [23].

Our results indicate that sleep quality is poorer in women and are consistent with earlier findings showing a higher prevalence of sleep difficulties among females. It supports the hypothesis that both gender and age are relevant determinants of sleep quality in the general population [22]. In line with previous studies, our findings also revealed that women reported higher TMD symptom scores than men. This aligns with existing literature indicating that women tend to develop TMD signs and symptoms more frequently, especially those related to pain, making gender a significant factor in TMD prevalence and severity [24].

In our present study, divorced and widowed respondents reported a lower quality of sleep than respondents who are single or married; hence, the loss of marriage may have adverse effects on sleep. An extensive population-based study supported this finding and found that divorced men and women were at greater odds of experiencing insomnia symptoms compared with their married counterparts, further demonstrating the importance of marital status in sleep health [25]. In our study, divorced and widowed persons described more severe TMD symptoms than single or married persons, indicating that marital disruption may be associated with greater pain. This is backed by a recent scoping review that showed a greater prevalence of TMD in divorced people, further supporting the role of marital status as a contributing sociodemographic variable [26]. Our findings are consistent with earlier research, which indicates that poorer quality sleep is associated with greater TMD symptom intensity, reinforcing the importance of evaluating and treating sleep in TMD treatment [19].

Limitations

Despite the merits of this study, several limitations should be noted. The first is that the cross-sectional design limits the capacity to make causal inferences regarding the association between sleep quality and TMD symptoms. Longitudinal studies would be required to clarify temporal and causal paths. Second, the use of self-report data for both sleep quality (PSQI) and TMD symptoms (FAI) introduces possible recall and reporting biases that can compromise the validity of the results. Third, the convenience sampling approach, while effective in recruiting a representative clinical population, may limit the generalizability of results to the broader community or across diverse cultural and healthcare settings outside Islamabad, Pakistan. Fourth, certain confounding factors related to the situation, like psychological distress, depression, or other comorbidities, were not explored in depth, and this may have affected sleep quality and TMD symptoms. One limitation of this study is that it did not collect detailed information on the type, dosage, or duration of medications that participants were taking. Although the participants were questioned on whether they were taking drugs at the time, the study did not ask about the characteristics of the medications they took. This may limit the ability to fully assess how certain medications, including their type and dosage, might influence sleep quality and TMD symptoms. More comprehensive data regarding drugs might enhance future research to establish these relationships more precisely. Another limitation is that the regression model used in this study was limited to a single predictor (sleep quality). Potential confounders such as tobacco use, caffeine consumption, anxiety, and depression were not included as covariates, which may have influenced the observed association between sleep quality and TMD symptoms. Finally, small subgroup sizes for some groups, such as widowed people or those who did not want to report their gender, may have compromised the power to detect significant differences within these groups.

Future direction

Future research should utilize longitudinal designs in investigating how sleep disturbances and TMD symptoms interact with each other over time and discovering potential causal mechanisms. The external validity of results would be enhanced by having larger and more diverse populations from various regions and cultures included in the sample. Studying patient sleep with objective tests such as polysomnography or actigraphy could help find out more about sleep problems in TMD cases. Besides, future studies must clarify the impact of factors such as stress, anxiety, depression, and lifestyle on people with sleep-related TMD. Approaching the treatment of sleep and TMD by using multidisciplinary strategies would help patients obtain better results. Further examination of marital status, gender identity, and age could locate demographic moderators that can guide treatment strategies.

## Conclusions

It was found that good sleep quality is strongly linked to better control of TMD symptoms among adults, suggesting that TMD patients frequently suffer from sleep disorders. Analysis of the results shows that differences in age, gender, and marital status may impact this association, and these differences are significant between groups. Due to these findings, including sleep quality in standard TMD assessments is now more important. Managing TMD, as well as sleep issues, follows a multidisciplinary approach to get better treatment and more positive results for patients. The results support ongoing research on the relationship between sleep and temporomandibular disorders in adults.

## Figures and Tables

**Figure 2:**
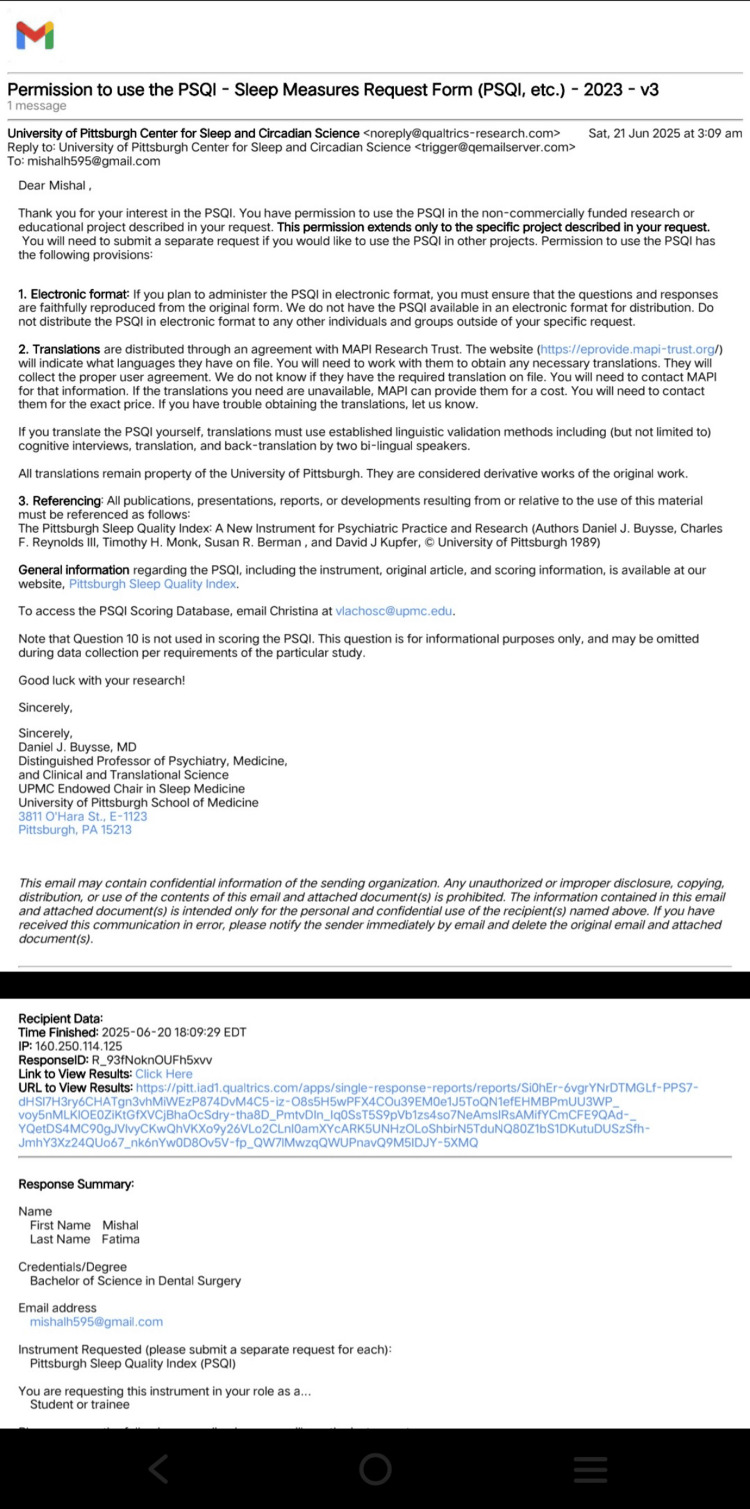
Permission Letter for Use of the Pittsburgh Sleep Quality Index (PSQI) Formal permission granted by the University of Pittsburgh Center for Sleep and Circadian Science to use the PSQI in a non-commercial academic study. This approval is specific to the current project and is provided under non-commercial educational use. Citation: Buysse, D. J., et al. (1989). The Pittsburgh Sleep Quality Index: A new instrument for psychiatric practice and research. University of Pittsburgh.

**Figure 3:**
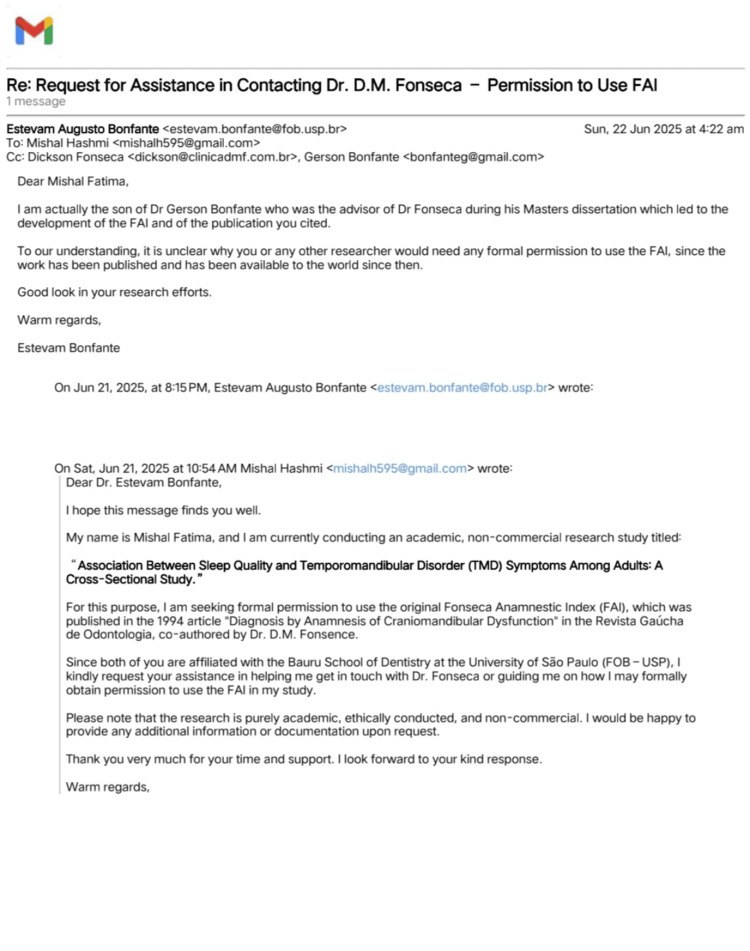
Confirmation Email for Use of the Fonseca Anamnestic Index (FAI) Email correspondence confirming that no formal permission is required to use the FAI for academic research, as it is publicly available through its original publication. Confirmation provided by Estevam Bonfante, affiliated with the original contributors to the scale’s development.

## Appendices

**Table 9:** Demographic Information Questionnaire

Items	Response
Age	-
Gender	-
Education level	-
Occupation	-
Marital Status	-
Do you have any known chronic illnesses?	-
Are you currently taking any medications?	-
Do you use tobacco or smoking products?	-
Do you consume caffeinated drinks (tea/coffee/energy drinks)?	-
